# Choosiness as a Predictor of Sexual (In)frequency in Single Heterosexual Adults

**DOI:** 10.1007/s10508-025-03160-z

**Published:** 2025-06-04

**Authors:** Henry S. G. Close, Lewis Nitschinsk, Brendan P. Zietsch, Fiona Kate Barlow

**Affiliations:** The School of Psychology, The University of Queensland, Brisbane, 4072 Australia

**Keywords:** Sexual frequency, Sexual infrequency, Choosiness, Partnered sexual activity, Heterosexual adults, Sexlessness

## Abstract

Increasing numbers of young men and women report celibacy or infrequent sex. In the present work, we consider “choosiness” as a potential correlate of partnered sexual activity for single people. We examined the tendency to be “choosy” in selecting a partner (i.e., choosiness) in two ways: choosiness based in reported essential characteristics in a partner (stated choosiness) and revealed by ratings of dating profiles (revealed choosiness). Results from a survey of 340 single, heterosexual, American participants (aged 18–40 years, 170 female) showed that choosiness relates to sexual frequency, but it depended on how it was measured. Stated choosiness was associated with more partnered sexual activity, whereas revealed choosiness was associated with engaging in less partnered sexual activity. These relationships remained significant even when controlling for pre-established correlates of sexual frequency, such as gender and self-rated attractiveness. This research provides a starting point for understanding choosiness and its role in single heterosexual adults’ sexual experience.

**Supplementary Information:**

The online version contains supplementary material available at 10.1007/s10508-025-03160-z.

## Introduction

Increasingly, young men and women report going for extended periods without having sex with a partner. For example, in 2018 almost 31% of US men aged 18–24 reported not having had sex in the past year, compared to approximately 19% in 2000 (Ueda et al., 2020), while the same reported experience doubled between 2000 and 2018 for men and women aged 25–34 (Ueda et al., 2020). In addition, in the USA, approximately 15% of people born in the 1990s are virgins in their early 20s (Twenge & Park, 2019; Twenge et al., 2017). Similar trends are evident in the UK, Australia, Germany, and Japan (Burghardt et al., 2020; de Visser et al., 2014; Ghaznavi et al., 2019; Herbenick et al., 2022; Wellings et al., 2019).

There is reason to take the amount of sex people are having seriously. Most individuals report wanting partnered sex, and a satisfying sex life is associated with health, longevity, well-being, and relationship satisfaction (Buczak-Stec et al., 2019; McNulty et al., 2016; Schmiedeberg et al., 2017; Schoenfeld et al., 2017), although note that not all people want or need partnered sex (Bogaert, 2015; Carvalho & Rodrigues, 2022; Higginbottom, 2024). In the present paper, we introduce choosiness (the tendency to be “picky” or discerning when it comes to potential partners; Johnstone et al., 1996; Shuker & Kvarnemo, 2021) as a factor to consider in understanding whether and how much single people engage in partnered sexual activity. We operationalize choosiness in two ways—stated choosiness (asking people to list how many non-negotiable traits they want in a potential partner) and revealed choosiness (using ratings of dating profiles). First, we provide some background to the extant literature on sexual activity.

One of the primary factors associated with partnered sexual activity is physical attractiveness (Amos & McCabe, 2015) and more attractive people tend to have more sexual partners and more frequent sexual activity (Amos & McCabe, 2017; Rhodes et al., 2005). Relatedly, one longitudinal study showed that body satisfaction in adolescence predicts sexual frequency in young adulthood (Kvalem et al., 2019). Women who are either other- or self-rated as more attractive tend to have more sex (Perilloux et al., 2013) and attractiveness is associated with more sexual activity in a qualitative female sample (Thomas et al., 2019). Unmarried and older people report more sexlessness (defined as no sexual partners in the past year) than do married and younger people (Kim et al., 2017; Træen et al., 2019).

### The Role of Choosiness in Partnered Sexual Frequency

Human sexual relationships—at least in modern, Western societies—typically involve mutual mate choice (Arnocky, 2018; Fletcher et al., 2015; Johnstone et al., 1996; Stewart-Williams & Thomas, 2013), in which both men and women are choosy about whom they partner with. Much of the research outlined above examines factors that might affect how able an individual is to attract a partner to have sex; less examined is the other side of the coin: whether an individual accepts the partner, which depends on a person’s choosiness. Therefore, choosiness might be a factor that affects partnered sexual frequency.

Choosy individuals may have stringent criteria about how a potential partner should look and act, and what sort of personality they should have. The causes and consequences of this variation are not well understood. Intuitively it seems likely that choosier individuals will have more difficulty finding a partner since acceptable partners will be fewer and of higher quality and thus more in demand from competitors. Consistent with this idea, singles cite being “too picky” as their most prominent reason for being single (Apostolou & Esposito, 2020), and people report choosiness as being the second most important difficulty that they face in moving to start a relationship (behind “fear of getting hurt”; Apostolou, 2021b).

On the other hand, though, choosiness is sometimes found to be associated with high mate value. In speed dating studies there is some evidence, though inconsistent, that more attractive individuals say yes to fewer speed dating partners than do less attractive individuals (Asendorpf et al., 2011; Kurzban & Weeden, 2005; Todd et al., 2007). Relatedly, people who perceive themselves as high mate value tend to be more selective in their partner choice (Arnocky, 2018; Csajbók & Berkics, 2022; Csajbók et al., 2023). Therefore, greater difficulties for choosier individuals may be offset or outweighed by higher mate value of those individuals. Consequently, we do not have a clear idea of whether to expect choosiness to be positively or negatively or not at all associated with the extent to which people do (or do not) have sex. The only directly relevant data comes from Apostolou (2021a), who found no association between stated choosiness and difficulty attracting a partner or length of singlehood. Singles who saw themselves as choosy were more likely to report preferring to be single, adding another dimension of complexity to the issue.

Apostolou’s (2021a) evidence, while informative, relies on self-reported choosiness. Studies of mate preferences have shown little if any relation between what people say they want in a partner (stated preferences) and the preferences revealed by their choices, such as in speed dating studies (revealed preferences; Eastwick et al., 2014; Schröder-Abé et al., 2016; Todd et al., 2007); a similar phenomenon might apply for choosiness. Further, caution is warranted when we consider explicit measures of self-reported choosiness given the stigma associated with being sexually or romantically undiscerning. There may be other reporting biases too. Individuals might be reporting their choosiness with reference to some imagined population norm or with reference to the standards they feel they should expect given their self-perceived mate value—this could lead to quite different responses depending on participants’ interpretation of the question. In all, there are several reasons why it would be valuable to use more sophisticated methods of assessing choosiness to test the association of choosiness with sexual frequency. In the present work we aim to do just this.

We also include in our sample single women as well as men. Some previous studies related to sexual activity, in particular abstinence, have focused exclusively on men (e.g., Irfan et al., 2020; Jaki et al., 2019). We believe this is an oversight, as many women report being unable to find a sexual or romantic partner (Eisenberg et al., 2009; Kim et al., 2017). There is reason to believe that the experience of partnered sexual activity for single people may differ between men and women, however. As women are on average choosier they are often understood as relationship “gatekeepers” who decide whether to begin a relationship by selecting or rejecting a potential partner (Kelly et al., 2015). Though this is of course a generalization, it means we might expect a stronger association between choosiness and the frequency of partnered sexual activity in women than in men—the latter’s sexual frequency being relatively more affected by overall attractiveness.

Finally, whether a person chooses to be single or not might also be associated with how choosiness is related to frequency of partnered sexual activity. Choosing to be single is associated with self-fulfillment and autonomy, whereas a lack of choice in being single is associated with regret and dissatisfaction (Girme et al., 2023; Kislev, 2020; Park & MacDonald, 2022; Stein, 1975; Timonen & Doyle, 2014). Research suggests that those who choose to be single are less motivated to seek sex and relationships than those who prefer to have a partner and are actively searching for someone, whether it be a meaningless fling or a committed relationship (Girme et al., 2016, 2023; Li & Kenrick, 2006; Regan et al., 2000). Therefore, choosiness and self-rated attractiveness may be less relevant when it comes to predicting sexual frequency in people who choose to be single.

### The Present Study

From the literature reviewed above, several gaps are evident. First, recent research has highlighted a rise in sexual inactivity among young adults, yet little attention has been given to the role of choosiness in explaining this trend. Second, existing work on sexual frequency primarily focuses on factors that influence how attractive someone is to others, rather than whether they accept potential partners. Third, prior work on choosiness relies heavily on self-reported mating selectivity, which may be biased or inconsistent with actual behavior. Fourth, few studies have explored gender differences in choosiness or included both men and women, despite evidence that experiences of partnered sex may differ by gender. Fifth, the distinction between being single by choice versus circumstance also remains underexamined, even though it may shape how choosiness relates to sexual activity.

To address these gaps, in the present work we aim to investigate the association between choosiness and frequency/incidence of partnered sexual activity in single heterosexual male and female adults. We also assess previously identified correlates of sexual frequency (i.e., age, gender, and self-rated attractiveness) to determine whether choosiness is associated with sexual frequency after accounting for them. The research is primarily exploratory, but we do have some expectations based on previous research and theory.

First, considering the overall picture of the research on choosiness discussed above, we tentatively predict that choosy (relative to non-choosy) people will report having less frequent partnered sexual activity (H1). Second, we predict that participants who see themselves as more attractive (H2) will have more partnered sex. Third, we will test in an exploratory fashion for interactions between each choosiness variable, gender, and choice in being single (exploratory H3).

## Method

### Participants

A priori power analysis via G*Power (Faul et al., 2009) with power set at 0.80 revealed that a sample of roughly 395 participants was required to detect small effects. We therefore aimed to collect data from over 400 participants. A total of 408 participants from the USA clicked on the study via online paid participant research platform Prolific. Eligible participants were heterosexual, single men and women, aged between 18 and 40 (Final *N* = 340). The average age of the final sample was 25.09 years (SD = 5.60). We selected this age range as it is a societally normative time that people often seek to form sexual relationships, making it a particularly relevant time to study sexual frequency (Træen et al., 2019; Ueda et al., 2020).

To assess whether people were single we asked, “what is your relationship status?” with response options including “single”, “in a relationship”, “married”, or “other”. Only participants who self-identified as single were included in our sample. Participants were able to indicate different reasons for their single status. The most common reason for both men (58%) and women (78%) was “I haven’t found someone I like.” Additionally, 28% of men and 47% of women believed they were single because they were too choosy. For descriptive statistics of all reasons why people believed they were single see Table 1.

**Table 1:** Frequency for self-described reasons for being single for women and men

Reasons for being single	Women (%)	Men (%)
I haven’t found someone I like	78.2	58.8
I haven’t found someone that likes me	50.6	41.2
I want to be free to do what I want	39.4	35.3
I’m too busy	36.5	27.6
I have a low sex drive	5.9	4.1
I suffer from trauma due to sexual abuse	7.1	2.4
Religious reasons	7.1	6.5
I experience sexual problems	2.4	2.4
I have recently been rejected	4.7	8.8
I have been rejected too many times	7.1	5.9
I’m too choosy/picky	47.6	28.2
I’m heartbroken	15.9	11.8
I’m too depressed	19.4	21.2
I’m too socially isolated	40.6	37.6
My self-esteem is too low	37.1	30.0
I think I am ugly	27.1	24.7
I have a disability	2.9	2.9
I’m too shy	40.6	38.2
I’m too nervous	39.4	38.8
Other mental health reason	6.5	6.5
I’ve had bad experiences from past relationships	28.2	12.9
I’m too short	2.4	2.9
I’m too overweight/too fat	22.9	20.6
I have experienced sexual abuse/trauma	7.6	1.2
I just prefer it	21.2	12.9
I am emotionally unavailable	22.9	16.5
Other	5.9	4.7

Self-described choosiness as a reason for being single was not associated with either stated choosiness (*r* = .06) revealed choosiness (*r* = .10), average sexual frequency (*r* = −.07), frequency of most frequent sexual activity (*r* = −.08), or whether participants had any sexual activity in the past year (*r* = −.02)

### Measures

Below we outline all measures that are included in the main results of this manuscript. For additional measures that we have not included in the final paper, please refer to the supplemental materials.

#### Self-Rated Attractiveness

Participants were asked to separately rate their own facial attractiveness, bodily attractiveness, personality attractiveness, and overall attractiveness, on a 7-point scale (1 = not at all, 7 = extremely). These items were adapted from past work (Lee et al., 2020; Perilloux et al., 2012), and were averaged together to form a reliable scale, with higher scores indicating higher levels of self-rated attractiveness (α = 0.85).

#### Stated Choosiness

Participants viewed 12 traits each with three descriptors (e.g., “cultivated—has good manners, neat, polite”; Schwarz & Hassebrauck, 2012). We asked participants to think about their ideal partner, asking them, “Is this trait essential?” (yes/no), consistent with the satisficing approach to initial mate choice, where people reject partners who do not meet a minimum standard rather than exhaustively seeking someone to fit one’s ideals (Joel & MacDonald, 2021; Long & Campbell, 2015). How many qualities an individual sees as essential can then be used as an index of choosiness (the more essential qualities, the higher the level of choosiness). While these latter preferences are stated and not revealed, the measure does not explicitly mention choosiness or selectiveness, and thus avoids social desirability concerns that may be more evident in explicit measures. Note that while this measure does not involve people explicitly stating how choosy they are, it does involve people explicitly stating non-negotiables they require in a partner. Therefore, for ease of reading, we refer to this variable as stated choosiness (while recognizing that people are not explicitly stating how choosy they are).

#### Revealed Choosiness

**Fig. 1:**
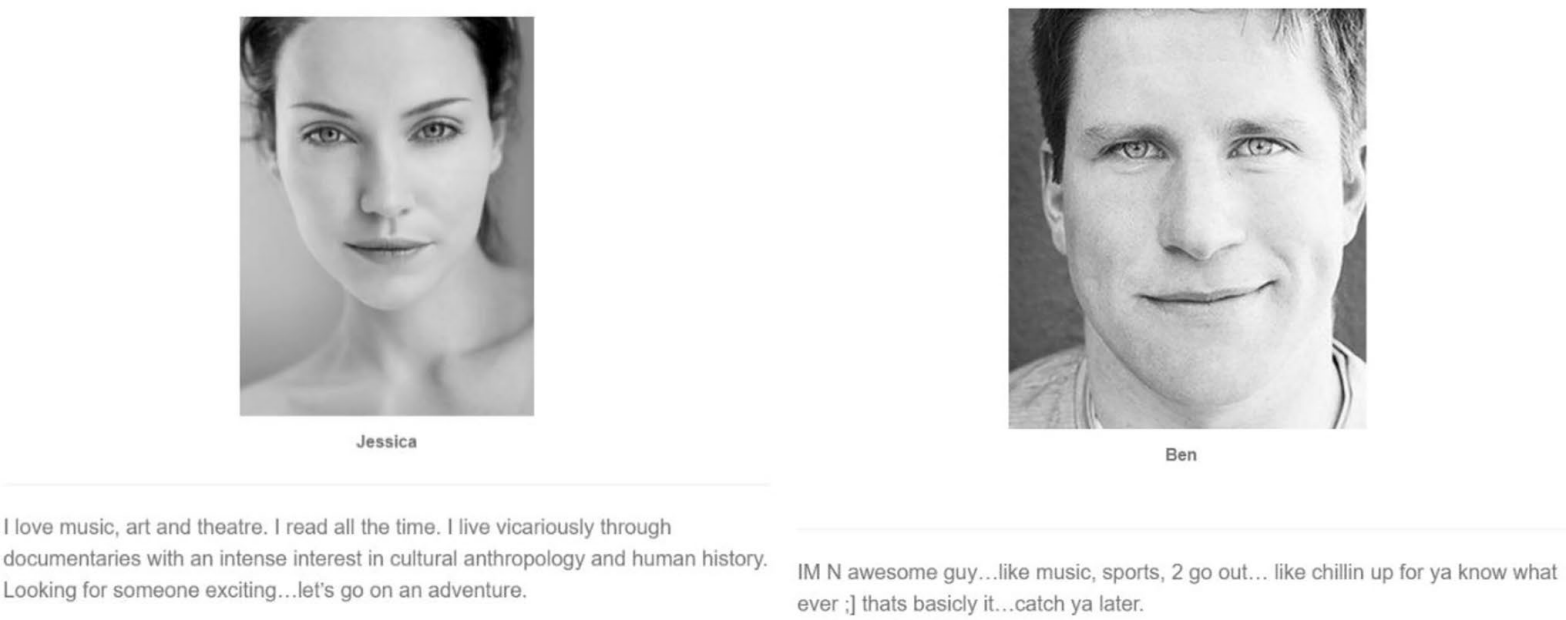
Example dating profiles. *Note*: Facial attractiveness and intelligence were randomized. These profiles used 20 images of men and women, each taken from stock image websites (e.g., http://www.gettyimages.com) along with and statements ostensibly written by the person in the image (though actually written by Lee et al., 2013)

To operationalize revealed choosiness, we first exposed people to dating profiles and asked them to indicate whether they found each profiled person attractive. This approach mimics the type of choosiness assessed in speed dating studies (e.g., Asendorpf et al., 2011; Finkel et al., 2007; Luo & Zhang, 2009; Schröder-Abé et al., 2016). Participants viewed 24 opposite-sex profiles (women rating profiles of men, and men rating profiles of women; see Fig. 1 for examples) taken from Lee et al. (2013) and were asked to answer the following questions: “Do you find (insert name) attractive?” (yes/no), “Could you see (insert name) as a romantic partner?” (yes/no) and “How willing would you be to go on a date with (insert name)?” (1 = not willing, 5 = very willing). We standardized all measures and averaged the responses. High scores indicated higher levels of revealed choosiness (α = 0.87).

#### Other Variables

For choice in being single, we asked participants to describe their single status, picking between one of two options: “I would prefer to have a partner” (0) or “I choose to be single” (1). Binary gender was coded as male (1) and female (0).

#### Frequency of Partnered Sex

We wanted to comprehensively assess the extent to which people had engaged in partnered sex over the last year. We initially created a 9-item scale to measure sexual activity in the past year. Participants rated how often they had participated in various sexual activities on a 5-point scale (not at all, rarely, somewhat, fairly often, often). These items included: penetrative sex, kissing, fondling, oral sex, clothes-on sex (e.g., “dry humping”), anal play (e.g., “rimming”), mutual masturbation, hand-to-genital contact (e.g., “fingering”), and other.

We created several measures using this scale. First, we averaged all items together to create a measure of average sexual frequency and variety (i.e., this measure assessed how frequently, on average, participants engaged in a variety of partnered sexual acts). Second, we took the absolute maximum score across all items to create a measure of sexual frequency unrelated to variety of sexual activities (i.e., this measure assessed how frequently, on average, participants engaged in any partnered sexual activity). Third, we created a dichotomous measure assessing whether participants had engaged in any sexual activity in the past year. We also created a dichotomous measure for engagement in penetrative sex in the past year, and whether participants reported being a virgin. Results for these latter two measures are included in the supplemental online materials.

### Data Analysis

We calculated bivariate correlations between all assessed variables. We then ran a series of multiple regressions to assess the effects of choosiness on sexual frequency. As described above, three dependent variables were assessed: average partnered sexual frequency, frequency of most frequently engaged in partnered sexual act, and whether participants had engaged in any sexual activity in the past year. For each regression stated and revealed choosiness were included as predictors at Step 1. At Step 2, age, gender, and why participants were single (i.e., by choice or not) were included as predictors. At Step 3, self-rated attractiveness was included as a predictor.

Finally, we conducted a series of exploratory moderated regression analyses. Expanding on the same models above, we included six interaction terms (gender*stated choosiness, gender*revealed choosiness, gender*self-rated attractiveness, single by choice*stated choosiness, single by choice*revealed choosiness, and single by choice*self-rated attractiveness).

## Results

### Descriptive Analyses

Thirty-six point six percent of our sample reported being virgins, 36.2% reported having no partnered sexual activity at all in the past year, and 56.1% reported having not had penetrative sex in the past year. Further descriptive statistics and bivariate correlations are shown in Table 2. Stated choosiness was positively associated with self-rated attractiveness, all sexual frequency measures and being female. Revealed choosiness was negatively associated with age, self-rated attractiveness, and all sexual frequency measures. Stated and revealed choosiness were not correlated.

**Table 2:** Means, standard deviations, and bivariate correlations for focal variables

	Range	M (SD)	1	2	3	4	5	6	7	8
1. Age	18–40	25.09 (5.60)								
2. Gender	0–1	0.50 (0.50)	−.01							
3. Single by choice	0–1	0.42 (0.49)	.10	.03						
4. Stated choosiness	1–12	7.25 (2.05)	−.01	−.16**	.01					
5. Revealed choosiness	− 2.46–2.38	0.00 (0.89)	−.19***	−.00	−.09	.01				
6. Self-rated attractiveness	1–7	4.28 (1.12)	.07	−.08	−.06	.31***	−.18**			
7. Average sexual frequency	0–3.78	0.70 (0.85)	.05	.07	−.00	.24***	−.19***	.34***		
8. Sexual frequency of most frequent sexual act	0–4	1.44 (1.37)	.04	.06	.03	.23***	−.22***	.37***	.88***	
9. Any sexual activity in the past year	0–1	0.64 (0.48)	.08	.06	.08	.17**	−.16**	.30***	.62***	.79***

** < .01. *** < .001. Gender is coded as 0 = female, 1 = male. Single by choice is coded as 0 = ”I prefer to have a partner”, 1 = ”I choose to be single”. Any sexual activity in the past year is coded as 0 = no sexual activity, 1 = sexual activity. Revealed choosiness is a standardized measure comprised of whether they find each person attractive or not, their willingness to date the person, and whether they could see the person as a romantic partner or not

### Regressions

For complete regression models see Table 3. We found that stated choosiness was positively associated, and revealed choosiness was negatively associated with average sexual frequency and frequency of most frequent sexual act. These associations remained significant when controlling for age, whether participants were single by choice, gender, and self-rated attractiveness. We also found that stated choosiness was positively associated, and revealed choosiness was negatively associated with engaging in any sexual activity in the past year (i.e., being sexless or celibate). However, the relationship for stated choosiness became marginally significant (*p* = .051), and the relationship for revealed choosiness just significant (*p* = .049) when self-rated attractiveness was included in the model, while also controlling for age, single by choice, and gender. Finally, in all models self-rated attractiveness was a positive predictor of partnered sexual activity. Being male was a positive predictor of average sexual frequency and frequency of most frequent sexual act.

**Table 3:** Linear models for stated choosiness, revealed choosiness, age, whether participants were single by choice, gender, and self-rated attractiveness on average sexual frequency, frequency of most frequent sexual act, and whether participants had engaged in any sexual activity in the past year

Panel A: average sexual frequency	Model 1			Model 2			Model 3			
	B	SE	95% CI	B	SE	95% CI	B	SE	95% CI	*r* (sr)
(Intercept)	0.00	0.05	− 0.10–0.10	0.00	0.05	− 0.10–0.10	0.00	0.05	− 0.10–0.10	
Stated choosiness	0.24***	0.05	0.14–0.34	0.26***	0.05	0.16–0.36	0.17**	0.05	0.07–0.28	.18 (17)
Revealed choosiness	− 0.20***	0.05	− 0.31– − 0.10	− 0.20***	0.05	− 0.30– − 0.09	− 0.15**	0.05	− 0.25– − 0.04	−.16 (−.15)
Age				0.02	0.05	− 0.08–0.12	0.01	0.05	− 0.10–0.11	.00 (.00)
Single by choice				− 0.03	0.05	− 0.13–0.08	0.01	0.05	− 0.10–0.10	−.01 (−.00)
Gender				0.11*	0.05	0.01–0.22	0.12*	0.05	0.02–0.22	.12 (.13)
Self-rated attractiveness							0.27***	0.05	0.17–0.38	.26 (.25)

Panel B: frequency of most frequent sexual act	Model 1			Model 2			Model 3			
	B	SE	95% CI	B	SE	95% CI	B	SE	95% CI	*r* (sr)
(Intercept)	− 0.00	0.05	− 0.10–0.10	− 0.00	0.05	− 0.10–0.10	− 0.00	0.05	− 0.10–0.10	
Stated choosiness	0.23***	0.05	0.13–0.34	0.25***	0.05	0.15–0.35	0.15**	0.05	0.05–0.26	.16 (.15)
Revealed choosiness	− 0.23***	0.05	− 0.33– − 0.13	− 0.22***	0.05	− 0.32– − 0.12	− 0.17**	0.05	− 0.27– − 0.07	−.19 (−.16)
Age				0.01	0.05	− 0.10–0.11	− 0.01	0.05	− 0.11–0.09	−.01 (−.01)
Single by choice				0.01	0.05	− 0.09–0.11	0.03	0.05	− 0.07–0.13	.03 (.03)
Gender				0.10	0.05	− 0.01–0.20	0.11*	0.05	0.01–0.20	.11 (.11)
Self-rated attractiveness							0.30***	0.05	0.20–0.41	.29 (.28)

Panel C: any sexual activity in the past year	Model 1			Model 2			Model 3			
	Odds ratio	SE	95% CI	Odds ratio	SE	95% CI	Odds ratio	SE	95% CI	*r* (sr)
(Intercept)	0.47	0.20	0.20–1.09	0.12*	0.10	0.02–0.62	0.02***	0.02	0.00–0.12	
Stated choosiness	1.21**	0.07	1.08–1.36	1.23**	0.07	1.09–1.39	1.14	0.07	1.00–1.29	.10 (.09)
Revealed choosiness	0.66**	0.09	0.50–0.85	0.68*	0.09	0.52–0.89	0.76*	0.11	0.57–1.00	−.11 (−.10)
Age				1.02	0.02	0.98–1.07	1.02	0.02	0.98–1.07	.04 (.04)
Single by choice				1.32	0.32	0.83–2.12	1.52	0.38	0.93–2.50	.08 (08)
Gender				1.48	0.35	0.93–2.37	1.56	0.39	0.96–2.55	.09 (.10)
Self-rated attractiveness							1.83***	0.22	1.38–2.25	.25

*N* = 340, CI = Confidence Interval, SE = Standard Error, *r* = partial correlation, sr = semi-partial correlation. Single by Choice: 0 = “I prefer to have a partner”, 1 = “I choose to be single”. Gender: 0 = Female, 1 = Male

### Exploratory Moderators

Full model results for each moderated multiple regression are included in the supplemental materials. Interactions were largely non-significant; gender did not moderate the association between either type of choosiness and partnered sexual activity, and choice in being single did not moderate the association between stated choosiness and outcome variables. In all models, however, we find a significant interaction between revealed choosiness and whether or not participants indicated that they were single by choice. For people who would have preferred to be in a relationship (i.e., did not report being single by choice) we found a negative relationship between revealed choosiness and average sexual frequency (estimate = −0.22, *p* < .001), frequency of most frequency sexual act (estimate = −0.41, *p* < .001), and any sexual activity in the past year (estimate = −0.58, *p* < .001). However, for people who were single by choice these relationships were not significant (*p*s > .05).

## Discussion

The proportion of men and women reporting going for long stretches without sexual contact has recently increased (Burghardt et al., 2020; de Visser et al., 2014; Eisenberg et al., 2009; Ghaznavi et al., 2019; Herbenick et al., 2022; Irfan et al., 2020; Kim et al., 2017; Ueda & Mercer, 2019; Ueda et al., 2020; Wellings et al., 2019), and a nascent body of work aims to understand this phenomenon (e.g., Apostolou, 2021a, 2021b; Apostolou & Esposito, 2020). To date, however, the research on sexual frequency has primarily focused on factors that might make people unappealing partners without fully considering how people’s active choice in partners might be associated with sexual frequency. In the present work we aimed to address this issue, examining how choosiness is associated with sexual (in)frequency, over and above self-rated attractiveness. We further investigated whether any associations between choosiness and sexual frequency might differ depending on whether participants were men or women, and whether participants (who were all single) were single by choice or would have preferred to have a partner. Overall, our results suggest that choosiness is an important factor for understanding partnered sexual frequency, but the relationship is complex and depends on how we operationalized choosiness.

First, and in line with our tentative hypothesis (H1) we found that people who were choosier when it came to rating dating profiles (revealed choosiness) reported having had fewer sexual encounters in the last year. This was only the case, however, for participants who would have preferred to be in a relationship (H3). This result is consistent with the idea that choosy people tend to refuse potential partners that they regard as below their standards, as well as pursuing potential partners that are “out of their league” (Penke et al., 2008; Spielmann et al., 2013, 2020). When participants indicated that they were single by choice; however, there was no association between revealed choosiness and frequency of engagement in partnered sexual activity.

The meaning of this latter finding requires interrogation. It may, on the one hand, indicate that revealed choosiness is only a negative predictor for people who are seeking relationships, but alternatively may reflect our measure of revealed choosiness. In our study we asked participants about the extent to which they were attracted to, would be open to dating, and could consider a relationship with, people in dating profiles. Two of these indices (those focusing on dating and a relationship) are perhaps mostly or even only relevant to those who want to be in a relationship. It is possible that choosiness solely based on looks or sexual attraction would show a different pattern, perhaps being particularly relevant to people seeking only casual sex (Vranken et al., 2024). Future work should consider further breaking down indices of choosiness.

The importance of considering multiple indices of choosiness is underscored when looking at the traits people listed as essential in a romantic partner. Here, participants were choosier when they listed more traits as essential (stated choosiness), but when looking at this index of choosiness the pattern was reversed: stated choosiness was associated with having more sex. This pattern was consistent when looking at sexual frequency both considering and excluding sexual variety, as well as when considering sexlessness as a binary construct. While we asked participants about traits required in an ideal partner, associations were not moderated by whether people wanted a relationship (vs. were single by choice; gender also didn’t moderate; refer to H3). So how do we understand the finding that people who stated that their ideal partner had to have a complex list of traits had more, rather than less, sexual activity?

One possibility is that people who see themselves as high in stated choosiness may also communicate this to potential partners—making those potential people feel more special and thus more likely to engage in a sexual relationship. This possibility is consistent with literature showing that people who are discerning and selective are rated as more attractive potential mates (Asendorpf et al., 2011; Kurzban & Weeden, 2005; Park & MacDonald, 2022). We also need to acknowledge that people who reported more stated choosiness also tended to rate themselves as more attractive than people who were lower in stated choosiness. This pattern is consistent with literature suggesting that choosiness reflects high mate value, and that choosy individuals’ pickiness is partially justified by their higher attractiveness and popularity (Asendorpf et al., 2011; Back et al., 2011; Kurzban & Weeden, 2005; Schröder-Abé et al., 2016). Again, however, the oppositive pattern was evident for revealed choosiness. Those who were choosier on this measure reported that they were less attractive than did less (revealed) choosy people. Perhaps this pattern in part explains the opposing associations between stated and revealed choosiness and sexual frequency as well as sexlessness; people who rated themselves as more attractive also reported having more partnered sexual activity (consistent with H2). Controlling for self-rated attractiveness did not eliminate the observed patterns however, and thus we need to look for additional explanations.

Returning to the unexpected positive association between stated choosiness and frequency of partnered sexual activity, it may be that people who can make declarative statements about what they want in a mate demonstrate confidence, which is often perceived as attractive (Bale & Archer, 2013; Buss, 2016; Murphy et al., 2015). People who know what they want may also have greater capacity to make informed decisions, which may mean that their navigation of sexual relationships is more successful. Finally, those who report more essential traits may also be those who are more interested and committed to the dating process relative to those who report fewer essential traits. Thus, those who report more essential traits may be more motivated to take steps to have a date with a partner and thus have more partnered sex.

It should be noted at this point that neither of our findings (that choosiness is both positively and negatively associated with sexual infrequency, depending on the way in which it is operationalized) corresponds to findings reported by Apostolou (2021a), who showed that self-described choosiness was unrelated to difficulty attracting a partner. Here, the differing measures are likely to be important. It is possible that when asked directly about choosiness social desirability might come into play, as there is a stigma attached to being undiscerning about potential mates. Indeed, we found that self-described choosiness was not correlated with either stated or revealed choosiness. This brings us to a larger point, that of how choosiness is measured. In the current work we include measures designed to reflect how we seek short- and long-term mates in the real world; one measure reflecting accepting potential mates on dating apps (revealed choosiness), and one measure reflecting pickiness in the additive combination of traits we see as non-negotiable in an ideal mate (stated choosiness).

It is not unusual for stated and revealed preferences to be unrelated to one another (e.g., in speed dating studies; Eastwick et al., 2014; Todd et al., 2007). Further confusing the issue, however, is the fact that neither measure was related to whether people saw themselves as choosy. We suspect that choosiness is a mixed phenomenon, broken down into whether people see themselves as choosy, as well as the extent to which they are choosy on dimensions reflecting appearance, character, sexual history, and so on. These different types of choosiness may not be associated with one another. A separate study on different types of choosiness, and how they relate to each other, would be beneficial in the study of choosiness and how it relates to mating success.

Moving on from measurement, we also want to take a moment to discuss the lack of moderation by gender. Theoretically it was possible that choosiness might be more predictive of sexual frequency for women than men, given that women are the choosier sex, and thus may have more latitude to express and act on their choices. Consistent with previous literature women in our sample showed more stated choosiness than men (Buss, 1989; Thomas et al., 2020; Walter et al., 2020). However, we found no evidence of moderation between gender and choosiness on sexual frequency. Gender differences are sometimes overstated (Stewart-Williams & Thomas, 2013; Ziogas et al., 2023) and so we want to take our lack of moderation seriously. In future work, it would be good to consider gender roles, as well as gender. Recent work reveals that adherence to traditional sexual scripts around female submission to men is associated with sexual dissatisfaction for some women (Bonell et al., 2022). Drawing on this work we suggest that it is possible that the association between choosiness and sexual frequency could vary for men and women, but only to the extent that they adhere to traditional gender roles.

### Strengths, Limitations, Implications, and Future Directions

In the above section we have discussed our findings, while also making suggestions about where the study could be improved and what might be fruitful areas of future research. Strengths, as well as additional limitations and future directions should also be considered. In terms of strengths, this study contributes to the literature by conducting novel research on the correlates of sexual frequency in single heterosexual adults, with an emphasis on two dimensions of choosiness. Our findings shed light on the correlates of sexual frequency as well as the characteristics of sexually isolated heterosexuals. Thirty-six percent of our participants were virgins, and the same percentage had not engaged in any sexual activity in the past year (note that the two groups were not colinear). Sexual isolation can have significant mental health implications including feelings of social inadequacy and unmet relational difficulties (e.g., Donnelly et al., 2001; Jaki et al., 2019). Our study suggests that fastidiousness in terms of potential partners on dating apps may be negatively related to sexual activity, whereas feeling choosy in terms of traits may be positively related to partnered sexual activity.

Such findings may be useful, especially for people who are wanting and desiring sexual activity. For singles seeking more sexual or romantic connection, the findings suggest that being clear and confident about one’s preferences (stated choosiness) may be linked with greater success, possibly because it signals self-assurance or commitment to dating. In contrast, being overly selective when evaluating dating profiles (revealed choosiness) is associated with reduced sexual activity, particularly among those who are not single by choice. This finding suggests that excessive pickiness in initial judgments may limit opportunities for connection, especially for those actively seeking a partner. For practitioners such as therapists, educators, and dating coaches, the findings underscore the value of helping clients distinguish between different forms of choosiness and align their expectations with their goals, whether casual or committed. Importantly, the study also points to the emotional challenges faced by involuntarily single individuals, for whom high choosiness may become a barrier rather than a strategy. At a broader level, the research emphasizes the need to recognize sexual inactivity as a growing and potentially impactful phenomenon. Relationship education efforts should consider not just safety and consent, but also how people navigate choice, self-perception, and motivation in an increasingly complex dating landscape.

Turning to additional limitations, our study was correlational and thus we can’t make any causal inferences. It could easily be, for example, that people low in self-rated attractiveness and people less sexually successful became bitter about dating, for example, and thus were less inclined to accept any potential match (i.e., were higher in what appeared to be revealed choosiness). Longitudinal work looking at choosiness and sexual connection would be particularly useful to establish causal precedence.

Our measure of sexual activity could also be improved. For example, although we know what types of sexual activity participants engaged in, we do not know what sexual activities they felt were most important and meaningful. It could well be the case that some participants, for example, wanted to engage in partnered penetrative sex specifically, but were unsuccessful in doing so. Future work should look at both sexual activity and sexual activity preference, clearly delineating between sexlessness and sexual frequency. Future research should also examine solo as well as partnered sexual activity. People who masturbate more may use it a substitute for partnered sex, as per the compensatory model (Das et al., 2009; Huang et al., 2022), or masturbation may increase partnered sexual satisfaction and/or desire, as per the complementary model (Fischer & Træen, 2022; Herbenick et al., 2023). With that said, our research was chiefly concerned with partnered sexual activity and the role of choosiness in selecting a partner for sex. We also note that the research on the association between masturbation and sexual frequency overall is mixed (Fischer & Træen, 2022; Herbenick et al., 2023), and one study showed little association between frequency of partnered sex and solo masturbation (Regnerus et al., 2017).

Finally, we suggest that larger follow-up studies should be conducted, in which sexlessness and sexual frequency is examined in men, women, and transgender and gender diverse people. Such studies would require large sample sizes to appropriately test for moderation. Similarly, in the present study we considered only single heterosexual participants. It will be important to look at predictors of sexual frequency for partnered people, as well as Lesbian, Gay, bisexual, and other sexual minority people.

## Conclusions

People who are single often understand their single status to be the result of choosiness. In the present study we asked whether that was the case when it came to participation in partnered sexual activity. Our study illuminates the complex relationship between choosiness and sexual frequency among single heterosexual adults. We found that individuals who were more discerning in a dating app-style measure tended to have fewer sexual encounters, but only if they desired a relationship. In contrast, those with a detailed list of essential traits in a partner report higher sexual activity. This result suggests that choosiness, when reflecting confidence and clear preferences, might enhance sexual engagement. Choosiness is clearly subjectively meaningful to people, and we encourage future researchers to investigate how it practically contributes to singledom, sexlessness, and engagement in partnered (and non-partnered) sexual activity.

## Supplementary Information

Below is the link to the electronic supplementary material.

Supplementary file1 (DOCX 34 kb)

## Data Availability

Data is available upon request from the last author for the purpose of checking results.

## References

[CR1] Amos, N., & McCabe, M. P. (2015). Conceptualizing and measuring perceptions of sexual attractiveness: Are there differences across gender and sexual orientation? *Personality and Individual Differences,**76*, 111–122.

[CR2] Amos, N., & McCabe, M. (2017). The importance of feeling sexually attractive: Can it predict an individual’s experience of their sexuality and sexual relationships across gender and sexual orientation? *International Journal of Psychology,**52*(5), 354–363. 26445925 10.1002/ijop.12225

[CR3] Apostolou, M., & Esposito, G. (2020). Singles’ reasons for being single: Empirical evidence from an evolutionary perspective. *Frontiers in Psychology,**11*, 746. 32435217 10.3389/fpsyg.2020.00746 PMC7218110

[CR4] Apostolou, M. (2021a). Involuntary singlehood and its causes: The effects of flirting capacity, mating effort, choosiness and capacity to perceive signals of interest. *Personality and Individual Differences,**176*, Article 110782.

[CR5] Apostolou, M. (2021b). What makes It difficult to start an intimate relationship: A taxonomy of the reasons. *Europe’s Journal of Psychology,**17*(2), 103–116. 35136432 10.5964/ejop.1852 PMC8768479

[CR6] Arnocky, S. (2018). Self-perceived mate value, facial attractiveness, and mate preferences: Do desirable men want it all? *Evolutionary Psychology,**16*(1). 10.1177/1474704918763271 10.1177/1474704918763271 PMC10480998 29534596

[CR7] Asendorpf, J. B., Penke, L., & Back, M. D. (2011). From dating to mating and relating: Predictors of initial and long-term outcomes of speed-dating in a community sample. *European Journal of Personality,**25*(1), 16–30.

[CR8] Back, M. D., Penke, L., Schmukle, S. C., Sachse, K., Borkenau, P., & Asendorpf, J. B. (2011). Why mate choices are not as reciprocal as we assume: The role of personality, flirting and physical attractiveness. *European Journal of Personality,**25*(2), 120–132.

[CR9] Bale, C., & Archer, J. (2013). Self-perceived attractiveness, romantic desirability and self esteem: A mating sociometer perspective. *Evolutionary Psychology,**11*(1), 68–84. 23353113 10.1177/147470491301100107 PMC10480979

[CR10] Bogaert, A. F. (2015). *Understanding asexuality*. Rowman and Littlefield.

[CR11] Bonell, S., Lee, H., Pearson, S., Harris, E., & Barlow, F. K. (2022). Benevolent sexism and the traditional sexual script as predictors of sexual dissatisfaction in heterosexual women from the US. *Archives of Sexual Behavior,**51*(6), 3063–3070. 35790615 10.1007/s10508-022-02318-3 PMC9363330

[CR12] Buczak-Stec, E., König, H. H., & Hajek, A. (2019). The link between sexual satisfaction and subjective well-being: A longitudinal perspective based on the German ageing survey. *Quality of Life Research,**28*, 3025–3035. 31264125 10.1007/s11136-019-02235-4

[CR13] Burghardt, J., Beutel, M. E., Hasenburg, A., Schmutzer, G., & Brähler, E. (2020). Declining sexual activity and desire in women: Findings from representative German surveys 2005 and 2016. *Archives of Sexual Behavior,**49*(3), 919–925. 31802290 10.1007/s10508-019-01525-9

[CR14] Buss, D. M. (1989). Sex differences in human mate preferences: Evolutionary hypotheses tested in 37 cultures. *Behavioral and Brain Sciences,**12*(1), 1–14.

[CR15] Buss, D. M. (2016). *The evolution of desire: Strategies of human mating*. Basic Books.

[CR16] Carvalho, A. C., & Rodrigues, D. L. (2022). Sexuality, sexual behavior, and relationships of asexual individuals: Differences between aromantic and romantic orientation. *Archives of Sexual Behavior,**51*(4), 2159–2168. 35334025 10.1007/s10508-021-02187-2

[CR17] Csajbók, Z., & Berkics, M. (2022). Seven deadly sins of potential romantic partners: The dealbreakers of mate choice. *Personality and Individual Differences,**186*, Article 111334.

[CR18] Csajbók, Z., Štěrbová, Z., Brewer, G., Cândea, C. A., De Backer, C. J. S., Fernández, A. M., Fisher, M. L., Garcia, J. R., Kruger, D. J., Massar, K., Oberzaucher, E., Quintelier, K. J. P., Van Gefen, R. E., Valentova, J. V., Varella, M. A. C., & Jonason, P. K. (2023). Individual differences in how desirable people think they are as a mate. *Archives of Sexual Behavior,**52*(6), 2475–2490. 37154879 10.1007/s10508-023-02601-x PMC10501943

[CR19] Das, A., Parish, W. L., & Laumann, E. O. (2009). Masturbation in urban China. *Archives of Sexual Behavior,**38*(1), 108–120. 17710524 10.1007/s10508-007-9222-z

[CR20] de Visser, R. O., Richters, J., Rissel, C., Badcock, P. B., Simpson, J. M., Smith, A. M., & Grulich, A. E. (2014). Change and stasis in sexual health and relationships: Comparisons between the first and second Australian studies of health and relationships. *Sexual Health,**11*, 505–509. 25377003 10.1071/SH14112

[CR21] Donnelly, D., Burgess, E., Anderson, S., Davis, R., & Dillard, J. (2001). Involuntary celibacy: A life course analysis. *Journal of Sex Research,**38*(2), 159–169.

[CR22] Eastwick, P. W., Luchies, L. B., Finkel, E. J., & Hunt, L. L. (2014). The predictive validity of ideal partner preferences: A review and meta-analysis. *Psychological Bulletin,**140*(3), 623–655. 23586697 10.1037/a0032432

[CR23] Eisenberg, M. L., Shindel, A. W., Smith, J. F., Lue, T. F., & Walsh, T. J. (2009). Who is the 40-year-old virgin and where did he/she come from? Data from the national survey of family growth. *Journal of Sexual Medicine,**6*(8), 2154–2161. 19493289 10.1111/j.1743-6109.2009.01327.x

[CR24] Faul, F., Erdfelder, E., Buchner, A., & Lang, A.-G. (2009). Statistical power analyses using G* Power 3.1: Tests for correlation and regression analyses. *Behavior Research Methods,**41*(4), 1149–1160. 19897823 10.3758/BRM.41.4.1149

[CR25] Finkel, E. J., Eastwick, P. W., & Matthews, J. (2007). Speed-dating as an invaluable tool for studying romantic attraction: A methodological primer. *Personal Relationships,**14*(1), 149–166.

[CR26] Fischer, N., & Træen, B. (2022). A seemingly paradoxical relationship between masturbation frequency and sexual satisfaction. *Archives of Sexual Behavior,**51*(6), 3151–3167. 35790612 10.1007/s10508-022-02305-8 PMC9255456

[CR27] Fletcher, G. J., Simpson, J. A., Campbell, L., & Overall, N. C. (2015). Pair-bonding, romantic love, and evolution: The curious case of homo sapiens. *Perspectives on Psychological Science,**10*(1), 20–36. 25910380 10.1177/1745691614561683

[CR28] Ghaznavi, C., Sakamoto, H., Yoneoka, D., Nomura, S., Shibuya, K., & Ueda, P. (2019). Trends in heterosexual inexperience among young adults in Japan: Analysis of national surveys, 1987–2015. *BMC Public Health,**19*, 355. 30955502 10.1186/s12889-019-6677-5 PMC6452514

[CR29] Girme, Y. U., Overall, N. C., Faingataa, S., & Sibley, C. G. (2016). Happily single: The link between relationship status and well-being depends on avoidance and approach social goals. *Social Psychological and Personality Science,**7*(2), 122–130.

[CR30] Girme, Y. U., Park, Y., & MacDonald, G. (2023). Coping or thriving? Reviewing intrapersonal, interpersonal, and societal factors associated with well-being in singlehood from a within-group perspective. *Perspectives on Psychological Science,**18*(5), 1097–1120. 36534959 10.1177/17456916221136119 PMC10475216

[CR31] Herbenick, D., Rosenberg, M., Golzarri-Arroyo, L., Fortenberry, J. D., & Fu, T. C. (2022). Changes in penile-vaginal intercourse frequency and sexual repertoire from 2009 to 2018: Findings from the national survey of sexual health and behavior. *Archives of Sexual Behavior,**51*(3), 1419–1433. 34799832 10.1007/s10508-021-02125-2 PMC8604196

[CR32] Herbenick, D., Fu, T. C., Wasata, R., & Coleman, E. (2023). Masturbation prevalence, frequency, reasons, and associations with partnered sex in the midst of the COVID-19 pandemic: Findings from a U.S. nationally representative survey. *Archives of Sexual Behavior,**52*(3), 1317–1331. 36575264 10.1007/s10508-022-02505-2 PMC9794105

[CR33] Higginbottom, B. (2024). The nuances of intimacy: Asexual perspectives and experiences with dating and relationships. *Archives of Sexual Behavior,**53*(5), 1899–1914. 38539031 10.1007/s10508-024-02846-0

[CR34] Huang, S., Niu, C., & Santtila, P. (2022). Masturbation frequency and sexual function in individuals with and without sexual partners. *Sexes,**3*(2), 229–243.

[CR35] Irfan, M., Hussain, N. H. N., Noor, N. M., Mohamed, M., & Ismail, S. B. (2020). Sexual abstinence and associated factors among young and middle-aged men: A systematic review. *Journal of Sexual Medicine,**17*(3), 412–430. 31955912 10.1016/j.jsxm.2019.12.003

[CR36] Jaki, S., De Smedt, T., Gwóźdź, M., Panchal, R., Rossa, A., & De Pauw, G. (2019). Online hatred of women in the Incels.me forum: Linguistic analysis and automatic detection. *Journal of Language Aggression and Conflict,**7*(2), 240–268.

[CR37] Joel, S., & MacDonald, G. (2021). We’re not that choosy: Emerging evidence of a progression bias in romantic relationships. *Personality and Social Psychology Review,**25*(4), 317–343. 34247524 10.1177/10888683211025860 PMC8597186

[CR38] Johnstone, R. A., Reynolds, J. D., & Deutsch, J. C. (1996). Mutual mate choice and sex differences in choosiness. *Evolution,**50*(4), 1382–1391. 28565695 10.1111/j.1558-5646.1996.tb03912.x

[CR39] Kelly, A. J., Dubbs, S. L., & Barlow, F. K. (2015). Social dominance orientation predicts heterosexual men’s adverse reactions to romantic rejection. *Archives of Sexual Behavior,**44*(4), 903–919. 25224507 10.1007/s10508-014-0348-5

[CR40] Kim, J. H., Tam, W. S., & Muennig, P. (2017). Sociodemographic correlates of sexlessness among American adults and associations with self-reported happiness levels: Evidence from the U.S. General Social Survey. *Archives of Sexual Behavior,**46*(8), 2403–2415. 28275930 10.1007/s10508-017-0968-7 PMC5889124

[CR41] Kislev, E. (2020). How do relationship desire and sociability relate to each other among singles? Longitudinal analysis of the pairfam survey. *Journal of Social and Personal Relationships,**37*, 2634–2650.

[CR42] Kurzban, R., & Weeden, J. (2005). HurryDate: Mate preferences in action. *Evolution and Human Behavior,**26*(3), 227–244.

[CR43] Kvalem, I. L., Træen, B., Markovic, A., & von Soest, T. (2019). Body image development and sexual satisfaction: A prospective study from adolescence to adulthood. *Journal of Sex Research,**56*(6), 791–801. 30260677 10.1080/00224499.2018.1518400

[CR44] Lee, A. J., Dubbs, S. L., Kelly, A. J., von Hippel, W., Brooks, R. C., & Zietsch, B. P. (2013). Human facial attributes, but not perceived intelligence, are used as cues of health and resource provision potential. *Behavioral Ecology,**24*(3), 779–787.

[CR45] Lee, A. J., Sidari, M. J., Murphy, S. C., Sherlock, J. M., & Zietsch, B. P. (2020). Sex differences in misperceptions of sexual interest can be explained by sociosexual orientation and men projecting their own interest onto women. *Psychological Science,**31*(2), 184–192. 31971873 10.1177/0956797619900315

[CR46] Li, N. P., & Kenrick, D. T. (2006). Sex similarities and differences in preferences for short-term mates: What, whether, and why. *Journal of Personality and Social Psychology,**90*(3), 468–489. 16594832 10.1037/0022-3514.90.3.468

[CR47] Long, M.L.-W., & Campbell, A. (2015). Female mate choice: A comparison between accept-the-best and reject-the-worst strategies in sequential decision making. *Evolutionary Psychology,**13*. 10.1177/1474704915594553 37924191 10.1177/1474704915594553 PMC10426838

[CR48] Luo, S., & Zhang, G. (2009). What leads to romantic attraction: Similarity, reciprocity, security, or beauty? Evidence from a speed-dating study. *Journal of Personality,**77*(4), 933–964. 19558447 10.1111/j.1467-6494.2009.00570.x

[CR49] McNulty, J. K., Wenner, C. A., & Fisher, T. D. (2016). Longitudinal associations among relationship satisfaction, sexual satisfaction, and frequency of sex in early marriage. *Archives of Sexual Behavior,**45*(1), 85–97. 25518817 10.1007/s10508-014-0444-6 PMC4472635

[CR50] Murphy, S. C., von Hippel, W., Dubbs, S. L., Angilletta, M. J., Wilson, R. S., Trivers, R., & Barlow, F. K. (2015). The role of overconfidence in romantic desirability and competition. *Personality and Social Psychology Bulletin,**41*(8), 1036–1052. 26055389 10.1177/0146167215588754

[CR51] Park, Y., & MacDonald, G. (2022). Single and partnered individuals’ sexual satisfaction as a function of sexual desire and activities: Results using a sexual satisfaction scale demonstrating measurement invariance across partnership status. *Archives of Sexual Behavior,**51*(1), 547–564. 34997399 10.1007/s10508-021-02153-y PMC8741568

[CR52] Penke, L., Todd, P. M., Lenton, A. P., & Fasolo, B. (2008). How self-assessments can guide human mating decisions. In G. Geher & G. Miller (Eds.), *Mating intelligence: Sex, relationships, and the mind’s reproductive system* (pp. 37–75). Lawrence Erlbaum Associates Publishers.

[CR53] Perilloux, C., Easton, J. A., & Buss, D. M. (2012). The misperception of sexual interest. *Psychological Science,**23*(2), 146–151. 22261567 10.1177/0956797611424162

[CR54] Perilloux, C., Cloud, J. M., & Buss, D. M. (2013). Women’s physical attractiveness and short-term mating strategies. *Personality and Individual Differences,**54*(4), 490–495.

[CR55] Regan, P. C., Levin, L., Sprecher, S., Christopher, F. S., & Gate, R. (2000). Partner preferences: What characteristics do men and women desire in their short-term sexual and long-term romantic partners? *Journal of Psychology & Human Sexuality,**12*(3), 1–21.

[CR56] Regnerus, M., Price, J., & Gordon, D. (2017). Masturbation and partnered sex: Substitutes or complements? *Archives of Sexual Behavior,**46*(1), 2111–2121. 28341933 10.1007/s10508-017-0975-8

[CR57] Rhodes, G., Simmons, L. W., & Peters, M. (2005). Attractiveness and sexual behavior: Does attractiveness enhance mating success? *Evolution and Human Behavior,**26*(2), 186–201.

[CR59] Schmiedeberg, C., Huyer-May, B., Castiglioni, L., & Johnson, M. D. (2017). The more or the better? How sex contributes to life satisfaction. *Archives of Sexual Behavior,**46*(2), 465–473. 27757732 10.1007/s10508-016-0843-y

[CR60] Schoenfeld, E. A., Loving, T. J., Pope, M. T., Huston, T. L., & Štulhofer, A. (2017). Does sex really matter? Examining the connections between spouses’ nonsexual behaviors, sexual frequency, sexual satisfaction, and marital satisfaction. *Archives of Sexual Behavior,**46*(2), 489–501. 26732606 10.1007/s10508-015-0672-4

[CR61] Schröder-Abé, M., Rentzsch, K., Asendorpf, J. B., & Penke, L. (2016). Good enough for an affair: Self-enhancement of attractiveness, interest in potential mates and popularity as a mate. *European Journal of Personality,**30*(1), 12–18.

[CR62] Schwarz, S., & Hassebrauck, M. (2012). Sex and age differences in mate-selection preferences. *Human Nature,**23*(4), 447–466. 22941269 10.1007/s12110-012-9152-x

[CR63] Shuker, D. M., & Kvarnemo, C. (2021). The definition of sexual selection. *Behavioral Ecology,**32*(5), 781–794. 34695172 10.1093/beheco/arab055 PMC8528540

[CR64] Spielmann, S. S., MacDonald, G., Maxwell, J. A., Joel, S., Peragine, D., Muise, A., & Impett, E. A. (2013). Settling for less out of fear of being single. *Journal of Personality and Social Psychology,**105*(6), 1049–1073. 24128187 10.1037/a0034628

[CR65] Spielmann, S. S., Maxwell, J. A., MacDonald, G., Peragine, D., & Impett, E. A. (2020). The predictive effects of fear of being single on physical attractiveness and less selective partner selection strategies. *Journal of Social and Personal Relationships,**37*(1), 100–123.

[CR66] Stein, P. J. (1975). Singlehood: An alternative to marriage. *The Family Coordinator,**24*(4), 489–503.

[CR67] Stewart-Williams, S., & Thomas, A. G. (2013). The ape that thought it was a peacock: Does evolutionary psychology exaggerate human sex differences? *Psychological Inquiry,**24*(3), 137–168.

[CR68] Thomas, H. N., Hamm, M., Borrero, S., Hess, R., & Thurston, R. C. (2019). Body image, attractiveness, and sexual satisfaction among midlife women: A qualitative study. *Journal of Women’s Health,**28*(1), 100–106. 30307808 10.1089/jwh.2018.7107 PMC6343186

[CR69] Thomas, A. G., Jonason, P. K., Blackburn, J. D., Kennair, L. E. O., Lowe, R., Malouff, J., Stewart-Williams, S., Sulikowski, D., & Li, N. P. (2020). Mate preference priorities in the East and West: A cross-cultural test of the mate preference priority model. *Journal of Personality,**88*(3), 606–620. 31494937 10.1111/jopy.12514

[CR70] Timonen, V., & Doyle, M. (2014). Life-long singlehood: Intersections of the past and the present. *Ageing and Society,**34*(10), 1749–1770.

[CR71] Todd, P. M., Penke, L., Fasolo, B., & Lenton, A. P. (2007). Different cognitive processes underlie human mate choices and mate preferences. *Proceedings of the National Academy of Sciences,**104*(38), 15011–15016. 10.1073/pnas.0705290104 PMC1986604 17827279

[CR72] Træen, B., Štulhofer, A., Janssen, E., Carvalheira, A. A., Hald, G. M., Lange, T., & Graham, C. (2019). Sexual activity and sexual satisfaction among older adults in four European countries. *Archives of Sexual Behavior,**48*(3), 815–829. 29987546 10.1007/s10508-018-1256-x

[CR73] Twenge, J. M., Sherman, R. A., & Wells, B. E. (2017). Sexual inactivity during young adulthood is more common among U.S. millennials and igen: Age, period, and cohort effects on having no sexual partners after age 18. *Archives of Sexual Behavior,**46*(2), 433–440. 27480753 10.1007/s10508-016-0798-z

[CR74] Twenge, J. M., & Park, H. (2019). The decline in adult activities among U.S. adolescents, 1976–2016. *Child Development,**90*(2), 638–654. 28925063 10.1111/cdev.12930

[CR75] Ueda, P., & Mercer, C. H. (2019). Prevalence and types of sexual inactivity in Britain: analyses of national cross-sectional probability survey data. *BMJ Open,**9*(10), e030708. 31662376 10.1136/bmjopen-2019-030708 PMC6830683

[CR76] Ueda, P., Mercer, C. H., Ghaznavi, C., & Herbenick, D. (2020). Trends in frequency of sexual activity and number of sexual partners among adults aged 18 to 44 years in the US, 2000–2018. *JAMA Network Open,**3*(6), Article e203833. 32530470 10.1001/jamanetworkopen.2020.3833 PMC7293001

[CR77] Vranken, I., Sumter, S., & Vandenbosch, L. (2024). A multi-method study examining the role of swiping on dating apps: Mate value preferences, sexual satisfaction, and need satisfaction with matches in emerging adults. *Archives of Sexual Behavior,**53*(7), 2547–2582. 38839703 10.1007/s10508-024-02891-9

[CR78] Walter, K. V., Conroy-Beam, D., Buss, D. M., Asao, K., Sorokowska, A., Sorokowski, P., Aavik, T., Akello, G., Alhabahba, M. M., Alm, C., Amjad, N., Anjum, A., Atam, C. S., Duyar, D. A., Ayebare, R., Batres, C., Bendixen, M., Bensafia, A., Bizumic, B., ... Zupančič, M. (2020). Sex differences in mate preferences across 45 countries: A large-scale replication. *Psychological Science*, *31*(4), 408–423. 10.1177/0956797620904154 32196435

[CR79] Wellings, K., Palmer, M. J., Machiyama, K., & Slaymaker, E. (2019). Changes in, and factors associated with, frequency of sex in Britain: Evidence from three national surveys of sexual attitudes and lifestyles (natsal). *British Medical Journal,**365*, 66. 10.1136/bmj.l1525 PMC6503462 31064762

[CR80] Ziogas, A., Habermeyer, E., Santtila, P., Poeppl, T. B., & Mokros, A. (2023). Neuroelectric correlates of human sexuality: A review and meta-analysis. *Archives of Sexual Behavior,**52*(2), 497–596. 32016814 10.1007/s10508-019-01547-3

