# Supplementary material for: Choosiness as a Predictor of Sexual (In)frequency in Single Heterosexual Adults
Source: Arch Sex Behav. 2025 Jun 4;54(6):2095–106. doi: 10.1007/s10508-025-03160-z (PMC12283433; doi:10.1007/s10508-025-03160-z)
Supplement: Supplementary file 1 — Supplementary file1 (DOCX 34 kb) [file 10508_2025_3160_MOESM1_ESM.docx]

**Supplemental Materials**

**Method**

**Main Study Demographics Measurement**

We measured the choice to be single with a “voluntarily single (I choose to be single)” or “involuntarily single” (I don’t choose to be single)” binary forced choice question. We measured the remaining main study variables using open-ended questions. Gender—“What is your gender?”, with response options available as “female”, “male” or “other” (text box provided). Sexual orientation—What is your sexual orientation?”, with response options including “heterosexual”, “homosexual”, “bisexual”, or “other” (text box provided). Last, relationship status—“What is your relationship status?”, with response options including “single”, “in a relationship”, “married”, or “other” (text box provided). Any participant responses that did not include male or female, heterosexual, and single, were excluded due to the focus of our study.

**Choosiness Measurement**

For stated choosiness, 12 traits with three descriptors were included (Schwarz & Hassebrauck, 2012): “Kind and Understanding (e.g., , affectionate, emotional, loving)”, “Dominant (e.g., self-confident, assertive, has a mind of their own)”, “Pleasant (e.g., pleasant, straightforward, friendly)”, “Intellectual (e.g., high level of education, educated, intelligent)”, “Wealthy and Generous (e.g., wealthy, rich, generous)”, “Physically attractive (e.g., good looks, sexy looks, attractive)”, “Cultivated (e.g., neat, has good manners, polite)”, “Humorous (e.g., witty, funny, humorous)”, “Sociable (e.g., adventurous, spontaneous, outgoing)”, “Creative (e.g., creative and musical) and Domestic (e.g., good cook and domestic)”, “Reliable (e.g., honest, faithful, reliable)”, “Similar (e.g., similar interests, similar opinions, similar ideas of a relationship)”.

We asked three questions, including, “Is this trait essential?”, referred to in our main paper. We also asked, “How important is this trait?” (1 = *unimportant* to 5 = *very important*) and looked at this second mate preference choosiness scale (i.e., trait importance, α = .70). This showed similar results but as we wanted to investigate what was essential for personality traits, we solely retained the essential trait question (i.e., yes or no).

**Self-rated Attractiveness Measurement**

We asked participants the following questions, “How attractive is your face?”, “How attractive is your body?”, “How attractive is your personality?”, “How attractive are you overall?” (Lee et al., 2020; Perilloux et al., 2012).

**Adolescent Experiences Measurement**

A novel 6-item measure included the following statements related to adolescent experiences (aged 13-17) with the opposite gender (i.e., male participants received statements about experiences with females; female participants received statements about experiences with males): “I kissed a boy/girl”, “I went on dates with boys/girls”, “I had a boyfriend/girlfriend”, “I talked with boys/girls frequently”, “I had penetrative sex”, “I had sexual contact and/or activity that was not penetrative sex (e.g., oral sex, fondling)”.

**Larger Survey Variables**

Note that we pre-registered the method of our data collection as well as exclusions. This pre-registration was part of a larger pre-registration for a fourth-year undergraduate student project (https://osf.io/mgy6n). The measures used in this study were taken from a larger survey that also included measures of:

- Height
- Weight
- Ethnicity
- Place of residence
- Native Language
- Highest level of education
- Study
- Employment
- Autism diagnosis
- Income
- Living with Parents
- Religion
- Social Class
- COVID-19 anxiety
- Self-Esteem (Robins et al., 2001)
- Social isolation (Hughes et al., 2004)
- Mental health (K6; Kessler et al., 2002)
- Rejection Sensitivity (Downey & Feldman, 1996)
- Contact quality (Barlow et al., 2012)
- Dating and relationship experiences (e.g., dating frequency, use of dating applications, longest relationship)
- Incel identity
- Sexual identity (i.e., asexual, aromantic)

***Reasons for being single (Apostolou, 2017)***

In addition to asking participants whether they chose to be single or not, we also asked whether they found the choice to be single a “satisfying personal choice” (on a scale of 1 = *not at all* to 7 = *completely*). Then, we asked the reasons for being single/not having sex/not having a romantic partner, including the following:

- I haven't found someone I like
- I haven’t found someone that likes me
- I want to be free to do what I want
- I’m too busy
- I have a low sex drive
- I suffer from trauma due to sexual abuse
- Religious reasons
- I experience sexual problems
- I have been recently rejected
- I have been rejected too many times
- I’m too choosy/picky
- I’m heartbroken
- I’m too depressed
- I’m too socially isolated
- My self-esteem is too low
- I think I’m ugly
- I have a disability
- I’m too shy
- I’m too nervous/anxious
- Other mental health reason
- I’ve had bad experiences from past relationships
- I'm too short
- I"m overweight/too fat
- I have experienced sexual abuse
- I just prefer it
- I’m emotionally unavailable
- Other (text entry)

**Results**

**Table A**

*Means, Standard Deviations, and Bivariate Correlations for adolescent experiences, penetrative sex, and virginity on all other variables*

|  | Adolescent Experiences | Penetrative Sex | Virginity |
| --- | --- | --- | --- |
| Adolescent Experiences |  |  |  |
| Penetrative Sex | 0.43^***^ |  |  |
| Virginity | -0.50^***^ | -0.68^***^ |  |
| Age | 0.09 | 0.19^***^ | -0.38^***^ |
| Gender^a^ | 0.28^***^ | 0.09 | -0.07 |
| Single by choice^b^ | 0.04 | 0.05 | -0.02 |
| Stated choosiness | 0.08 | 0.13^*^ | -0.05 |
| Revealed choosiness | -0.16^**^ | -0.13^*^ | 0.11^*^ |
| Self-rated attractiveness | 0.22^***^ | 0.27^***^ | -0.23^***^ |
| Average Sexual Frequency | 0.43^***^ | 0.70^***^ | -0.50^***^ |
| Frequency of Most Frequent Sexual Act | 0.42^***^ | 0.68^***^ | -0.49^***^ |
| Any Sexual Activity in the Past Year | 0.41^***^ | 0.67^***^ | -0.52^***^ |

**Table B**

*Linear models for stated choosiness, revealed choosiness, age, single by choice, gender, and self-rated attractiveness on whether or not participants had penetrative sex in the past year and on whether or not participants are a virgin.*

|  | **Model 1** | | | | | | **Model 2** | | | | **Model 3** | | |
| --- | --- | --- | --- | --- | --- | --- | --- | --- | --- | --- | --- | --- | --- |
| ***Panel A: Penetrative Sex*** | | | | | | | | | | | | | |
|  | | *Odds Ratio* | *SE* | *95% CI* | *Odds Ratio* | *SE* | | *95% CI* | *Odds Ratio* | *SE* | | *95% CI* | *r^2^* |
| (Intercept) | | 0.29 ^**^ | 0.12 | 0.13 – 0.65 | 0.02 ^***^ | 0.02 | | 0.00 – 0.10 | 0.00 ^***^ | 0.00 | | 0.00 – 0.03 |  |
| Stated Choosiness | | 1.15 ^*^ | 0.06 | 1.03 – 1.28 | 1.18 ^**^ | 0.07 | | 1.05 – 1.32 | 1.09 | 0.07 | | 0.97 – 1.23 | .08 |
| Revealed Choosiness | | 0.72 ^*^ | 0.09 | 0.56 – 0.92 | 0.78 | 0.10 | | 0.60 – 1.01 | 0.85 | 0.12 | | 0.65 – 1.11 | -.07 |
| Age | |  |  |  | 1.07 ^**^ | 0.02 | | 1.02 – 1.11 | 1.07 ^**^ | 0.02 | | 1.02 – 1.11 | .16 |
| Single by Choice | |  |  |  | 1.11 | 0.26 | | 0.71 – 1.75 | 1.23 | 0.29 | | 0.77 – 1.97 | -.04 |
| Gender | |  |  |  | 1.65 ^*^ | 0.39 | | 1.05 – 2.62 | 1.78 ^*^ | 0.43 | | 1.11 – 2.87 | .12 |
| Self-Rated Attractiveness | |  |  |  |  |  | |  | 1.63 ^***^ | 0.20 | | 1.29 – 2.09 | .22 |
|  | **Model 1** | | | | | | **Model 2** | | | | **Model 3** | | |
| ***Panel B: Virginity*** | | | | | | | | | | | | | |
|  | | *Odds Ratio* | *SE* | *95% CI* | *Odds Ratio* | *SE* | | *95% CI* | *Odds Ratio* | *SE* | | *95% CI* | *r^2^* |
| (Intercept) | | 0.85 | 0.35 | 0.37 – 1.92 | 166.76 ^***^ | 164.06 | | 25.71 – 1227.67 | 1119.15 ^***^ | 1298.04 | | 125.48 – 11971.50 |  |
| Stated Choosiness | | 0.95 | 0.05 | 0.85 – 1.06 | 0.91 | 0.06 | | 0.81 – 1.03 | 0.99 | 0.07 | | 0.87 – 1.12 | -.01 |
| Revealed Choosiness | | 1.32 ^*^ | 0.17 | 1.02 – 1.70 | 1.14 | 0.16 | | 0.87 – 1.51 | 1.04 | 0.15 | | 0.78 – 1.38 | .02 |
| Age | |  |  |  | 0.84 ^***^ | 0.02 | | 0.79 – 0.88 | 0.83 ^***^ | 0.02 | | 0.78 – 0.87 | -.36 |
| Single by Choice | |  |  |  | 1.10 | 0.28 | | 0.68 – 1.81 | 0.98 | 0.25 | | 0.59 – 1.63 | .01 |
| Gender | |  |  |  | 0.64 | 0.16 | | 0.39 – 1.04 | 0.59 ^*^ | 0.15 | | 0.36 – 0.99 | -.10 |
| Self-Rated Attractiveness | |  |  |  |  |  | |  | 0.61 ^***^ | 0.08 | | 0.47 – 0.78 | -.20 |

***Note*:** *N* = 340, *CI* = Confidence Interval, *SE* = Standard Error, *r^2^* = semi-partial correlation. Single by Choice: 0 = “I prefer to have a partner”, 1 = “I choose to be single. Gender: 0 = Female, 1 = Male.

**Table C**

*Linear models for stated choosiness, revealed choosiness, age, single by choice, gender, self-rated attractiveness and their interactions on average sexual frequency, frequency of most frequent sexual act, and whether or not participants had engaged in any sexual activity in the past year.*

|  | **Average Sexual Frequency** | | | **Frequency of Most Frequent Sexual Act** | | | **Any Sexual Activity in the Past Year** | | |
| --- | --- | --- | --- | --- | --- | --- | --- | --- | --- |
| *Predictors* | *B* | *SE* | *95% CI* | *B* | *SE* | *95% CI* | *Odds Ratios* | *SE* | *95% CI* |
| (Intercept) | 0.03 | 0.05 | -0.07 – 0.13 | 0.03 | 0.05 | -0.07 – 0.13 | 4.04*** | 8.42 | 0.07 – 243.64 |
| Age | 0.01 | 0.05 | -0.10 – 0.11 | -0.01 | 0.05 | -0.11 – 0.09 | 1.02 | 0.02 | 0.98 – 1.07 |
| Single By Choice | 0.01 | 0.05 | -0.09 – 0.11 | 0.04 | 0.05 | -0.05 – 0.14 | 0.39* | 0.51 | 0.03 – 4.75 |
| Self-Rated Attractiveness | 0.30*** | 0.05 | 0.19 – 0.40 | 0.32*** | 0.05 | 0.22 – 0.43 | 0.84*** | 0.33 | 0.39 – 1.85 |
| Stated Choosiness | 0.15** | 0.05 | 0.04 – 0.26 | 0.14* | 0.05 | 0.03 – 0.24 | 0.80 | 0.17 | 0.52 – 1.22 |
| Revealed Choosiness | -0.14** | 0.05 | -0.24 – -0.03 | -0.15** | 0.05 | -0.26 – -0.05 | 0.52 | 0.25 | 0.20 – 1.30 |
| Gender | 0.14** | 0.05 | 0.04 – 0.24 | 0.12* | 0.05 | 0.02 – 0.22 | 0.05* | 0.07 | 0.00 – 0.64 |
| Single by Choice *  Self-Rated Attractiveness | 0.01 | 0.05 | -0.10 – 0.12 | 0.06 | 0.05 | -0.04 – 0.16 | 1.39 | 0.37 | 0.83 – 2.37 |
| Single by Choice *  Stated Choosiness | 0.05 | 0.05 | -0.05 – 0.16 | 0.04 | 0.05 | -0.06 – 0.14 | 1.01 | 0.13 | 0.77 – 1.31 |
| Single by Choice *  Revealed Choosiness | 0.11* | 0.05 | 0.01 – 0.22 | 0.13* | 0.05 | 0.03 – 0.23 | 2.20* | 0.67 | 1.22 – 4.05 |
| Gender *  Self-Rated Attractiveness | 0.03 | 0.05 | -0.07 – 0.14 | 0.06 | 0.05 | -0.04 – 0.17 | 1.59 | 0.43 | 0.95 – 2.74 |
| Gender *  Stated Choosiness | 0.09 | 0.05 | -0.01 – 0.20 | 0.05 | 0.05 | -0.05 – 0.16 | 1.26 | 0.17 | 0.96 – 1.66 |
| Gender *  Revealed Choosiness | -0.02 | 0.05 | -0.12 – 0.08 | -0.00 | 0.05 | -0.10 – 0.10 | 1.05 | 0.31 | 0.58 – 1.87 |

***Note*:** *N* = 340, *CI* = Confidence Interval, *SE* = Standard Error. Single by Choice: 0 = “I prefer to have a partner”, 1 = “I choose to be single. Gender: 0 = Female, 1 = Male.

**References**

Apostolou, M. (2017). Why people stay single: An evolutionary perspective. *Personality and Individual Differences, 111*(1), 263-271.

<https://doi.org/10.1016/j.paid.2017.02.034>

Barlow, F.K., Paolini, S., Pedersen, A., Hornsey, M. J., Radke, H. R. M., Harwood, J., Rubin, M. and Sibley, C. G. (2012). The contact caveat: negative contact predicts increased prejudice more than positive contact predicts reduced prejudice. *Personality and Social Psychology Bulletin, 38* (12), 1629-1643.

<https://doi.org/10.1177/0146167212457953>

Downey, G., & Feldman, S. I. (1996). Implications of rejection sensitivity for intimate relationships. *Journal of Personality and Social Psychology, 70*(6), 1327–1343.

<https://doi.org/10.1037/0022-3514.70.6.1327>

Hughes, M. E., Waite, L. J., Hawkley, L. C., & Cacioppo, J. T. (2004). A Short Scale for Measuring Loneliness in Large Surveys: Results From Two Population-Based Studies. *Research on Aging, 26*(6), 655–672.

<https://doi.org/10.1177/0164027504268574>

Lee, A. J., Dubbs, S. L., Kelly, A. J., von Hippel, W., Brooks, R. C., & Zietsch, B. P. (2013). Human facial attributes, but not perceived intelligence, are used as cues of health and resource provision potential. *Behavioral Ecology, 24*(3), 779-787. <https://doi.org/10.1093/beheco/ars199>

Lee, A. J., Sidari, M. J., Murphy, S. C., Sherlock, J. M., & Zietsch, B. P. (2020). Sex differences in misperceptions of sexual interest can be explained by sociosexual orientation and men projecting their own interest onto women. *Psychological Science, 31*(2), 184-192. <https://doi.org/10.1177/0956797619900315>

Kessler, R. C., Andrews, G., Colpe, L. J., Hiripi, E., Mroczek, D. K., Normand, S. L., Walters, E. E., & Zaslavsky, A. M. (2002). Short screening scales to monitor population prevalences and trends in non-specific psychological distress. *Psychological medicine, 32*(6), 959-976. <https://doi.org/10.1017/S0033291702006074>

Perilloux, C., Easton, J. A., & Buss, D. M. (2012). The misperception of sexual interest. *Psychological Science, 23*(2), 146-151. <https://doi.org/10.1177/0956797611424162>

Robins, R. W., Hendin, H. M., & Trzesniewski, K. H. (2001). Measuring Global Self-Esteem: Construct Validation of a Single-Item Measure and the Rosenberg Self-Esteem Scale. *Personality and Social Psychology Bulletin, 27*(2), 151–161. <https://doi.org/10.1177/0146167201272002>

Schwarz, S., & Hassebrauck, M. (2012). Sex and age differences in mate-selection preferences. *Human Nature, 23*(4), 447-466. <https://doi.org/10.1007/s12110-012-9152-x>
